# Using Text Messaging in Long-Term Arthroplasty Follow-Up: A Pilot Study

**DOI:** 10.2196/resprot.6047

**Published:** 2017-05-16

**Authors:** Oliver Blocker, Alison Bullock, Rhidian Morgan-Jones, Adel Ghandour, James Richardson

**Affiliations:** ^1^ Department of Trauma and Orthopaedics University Hospital of Wales Cardiff and Vale University Health Board Cardiff United Kingdom; ^2^ Cardiff Unit for Research and Evaluation in Medical and Dental Education Cardiff University School of Social Sciences Cardiff University Cardiff United Kingdom; ^3^ Cardiff and Vale Orthopaedic Centre University Hospital Llandough Cardiff and Vale University Health Board Cardiff United Kingdom; ^4^ Orthopaedic Institute The Robert Jones and Agnes Hunt Orthopaedic Hospital National Health Service Foundation Trust Oswestry United Kingdom

**Keywords:** texting, text messages, short message service, patient outcome assessment, follow-up studies, arthroplasty

## Abstract

**Background:**

Patient-reported outcome measures (PROMs) and mobile technology have the potential to change the way patients are monitored following joint replacement surgery.

**Objective:**

The aim of this study was to determine the feasibility of text messaging to record PROMs in long-term follow-up of hip and knee arthroplasty. Our participants were 17 patients 2-years-plus post hip or knee arthroplasty attending clinic with a mobile telephone number on record.

**Methods:**

A simple PROM (Oswestry Very Short Form) was texted to the patient. Responses were compared to clinical, radiographic, and existing PROM findings. Patients were interviewed to discover their opinions on this use of texting.

**Results:**

A total of 11 patients engaged with the text messaging. Reasons for not engaging included wrong numbers, physical barriers, and lack of understanding. A total of 8 patients attending clinic allowed comparison of text messaging with clinical findings. The average age was 70 years. A total of 4 patient text messaging responses matched clinical and radiographic findings; 3 also matched PROM scores collected in clinic. The 3 patients with mixed responses had abnormal clinical, radiographic, or PROM findings. One patient’s text responses conflicted with clinical outcome. Analysis of patients’ views showed a generally positive opinion: patients were happy to communicate with surgeons by text. Practical problems, PROM limitations, and trustworthiness of texting were highlighted.

**Conclusions:**

Engaging with changing technology creates challenges for patients and health care professionals. Despite this, our results suggest text messaging is a promising way to communicate with arthroplasty patients. Earlier integration of text communication in the patient pathway may be important and needs further research.

## Introduction

In an ideal world, every patient who undergoes joint replacement surgery should be followed up for the remainder of the life of the prosthesis or the patient. Failure rates in modern implants are low, but revision surgery is demanding, expensive, and distressing for the patient. An increase in primary procedures potentially creates a large revision burden [1]. Previous work investigating arthroplasty follow-up using technology has looked at short-term follow-up (less than 1 year) [2] and Internet- and computer-based evaluation [3].

Long-term follow-up of all arthroplasty patients in clinic would probably outstrip the capacity of most orthopedic outpatient departments in the National Health Service (NHS). Patient-reported outcome measures (PROMs) are common in arthroplasty surgery [4]. PROMs may have the potential to transform health care by measuring outcomes, prioritizing treatment and reimbursement. There are challenges to widespread PROMs use: minimizing the time, cost, collection, and analysis of data and maximizing patient participation. The adaptation of technology and Medicine 2.0 principles could be a way of encouraging widespread use of PROMs [5]. Studies outside of orthopedic surgery have noted that simple, short message service (SMS) interventions are as effective as more complex ones in modifying patient behavior [6]. Remote collection of PROMs and their comparison to results collected in outpatient clinics has been assessed previously, finding no difference between remote and on-site scores [7].

Mobile technology has developed since the early 2000s from simple, 2-way pagers to smartphones and tablet computers using wireless networks [8]. In the United Kingdom, 93% of adults own and use a mobile phone and 63% own a smartphone. Text messages or SMS are a common form of communication; the average number of text messages sent per person, per month in the United Kingdom was 117 in 2014 [9]. The literature on text messaging in health interventions is generally positive. It highlights good acceptance and efficacy, but the evidence base is limited [10]. Studies have shown that text messaging is a valid method of reminding patients about outpatient appointments [11] and that patients up to 75 years old show confidence with reading messages [12]. The issue regarding age and mobile technology uptake is evolving. Mobile technology itself presents challenges to older patients but these are likely to be practical (such as poor dexterity and vision) rather than due to attitudes and perceptions [13]. Further, older patients of tomorrow will be more familiar with and reliant on mobile technology than the current older generation [14]. For the target population in this study (60 years and older), the success of communication using mobile technology depends on how it is adapted and tailored to their needs [15].

Text messaging is a basic form of mobile communication. If shown to be a feasible way of communicating with arthroplasty patients, it has the potential to decrease the outpatient burden, collect PROMs data, and extend communication with patients beyond existing capabilities. This study aimed to determine the feasibility of text messaging as a means of communicating PROMs with long-term arthroplasty patients. We intended this study to be the first step to further research into mobile technology and PROMs.

## Methods

We investigated how patient text message PROMs responses compared to their clinical and radiological findings, and we elicited patient opinions on communicating with their surgical team by text message. This project was approved by the Cardiff and Vale University Health Board (CAVUHB) Continuous Service Improvement group as a service evaluation on January 9, 2015. Permission was granted to perform a small-scale pilot study comparing a text messaging assessment to clinical evaluation of patients attending continuing outpatient care by their operating surgeon.

The Cardiff and Vale Orthopaedic Centre (CAVOC) currently uses an email-based PROM monitoring system (Amplitude Clinical, Worcestershire, UK) to collect patient-generated joint scores, standard health outcomes, and “family and friends” [16] measures (how likely the patient would be to recommend the hospital). This has been in place since January 2015 and has no long-term results yet, so for our pilot study the principal change was to replace email with a short PROM delivered and responded to by text message.

The sample population was patients of two arthroplasty consultants at CAVUHB (RMJ/AG) who were more than 2 years following a primary hip or knee joint replacement, were already attending a follow-up appointment, and had a mobile telephone number on the electronic patient record system.

Eligible patients were sequentially texted using a smartphone (Apple iPhone 3GS), 2 to 3 days prior to their clinic appointment, a message of introduction, explanation, reminder of their surgery and appointment, and consent to participate. Patients who responded were then sent the PROM, developed at the Robert Jones and Agnes Hunt Orthopaedic Hospital and named the Oswestry Very Short Form (VSF). It consists of 2 questions: “Are you happy with your joint replacement?” and “Would you have it done again,” with the responses “yes” or “no.” The principle of the VSF is to measure patient satisfaction (happiness). This measure provides 3 outcomes: a positive response (yes/yes), a negative response (no/no), and a mixed response (yes/no or no/yes). A positive response should indicate satisfaction; a negative, dissatisfaction with the joint replacement; and a mixed response would indicate a need to investigate further. Patient text message responses were then compared to the consultant surgeon’s assessment of them in clinic and their radiographic findings (signs of loosening or wear of the prosthesis). The patients also were assessed using existing, validated PROMs. The hip arthroplasty patients were assessed using the physician-derived Harris Hip Score (HHS) [17], and the knee arthroplasty patients completed the patient-derived Oxford Knee Score (OKS) [18]. As patients were unevenly distributed over 8 clinics spanning 2 months, inclusion was cumulative.

Data collected were anonymized and transposed from the survey tool to CAVUHB systems to ensure data security and confidentiality. Patient age, gender, prosthesis, surgeon, and year of surgery were recorded. The text messaging engagement and PROM responses were recorded as well as the PROM completed in clinic. Radiographs were analyzed by the consultant surgeon and recorded alongside the patient symptoms and signs in their clinic letter. After completion, the mobile device was wiped of all information.

For qualitative data collection, face-to-face survey techniques were used by the principal investigator (OB). The patients who attended were met on arrival to the outpatient clinic, where OB introduced himself as the author of the text messages, explained the study to the patients, and gained their consent to be interviewed during their visit to the clinic. The intention was to follow a semistructured interview guide, shown in Textbox 1.

**Semistructured interview guide.**

Interview questions

- Did you know we had your mobile phone number?
- What was your opinion on receiving the messages?
  - Prompt: Did you think it was appropriate to receive text messages from the Health Board?
  - Prompt: Were the messages clear and understandable?
  - Prompt: Did they allow you to express how you felt?
- What are your thoughts on receiving messages from us in the future?
- If you were unable to continue seeing your surgeon, would you be happy to use text messaging or other forms of communication?

Closed and open questions were followed by prompts to maintain a conversation and elicit meaningful opinions. The prompts were flexible and were not always used.

## Results

### Overview

**Figure 1:**
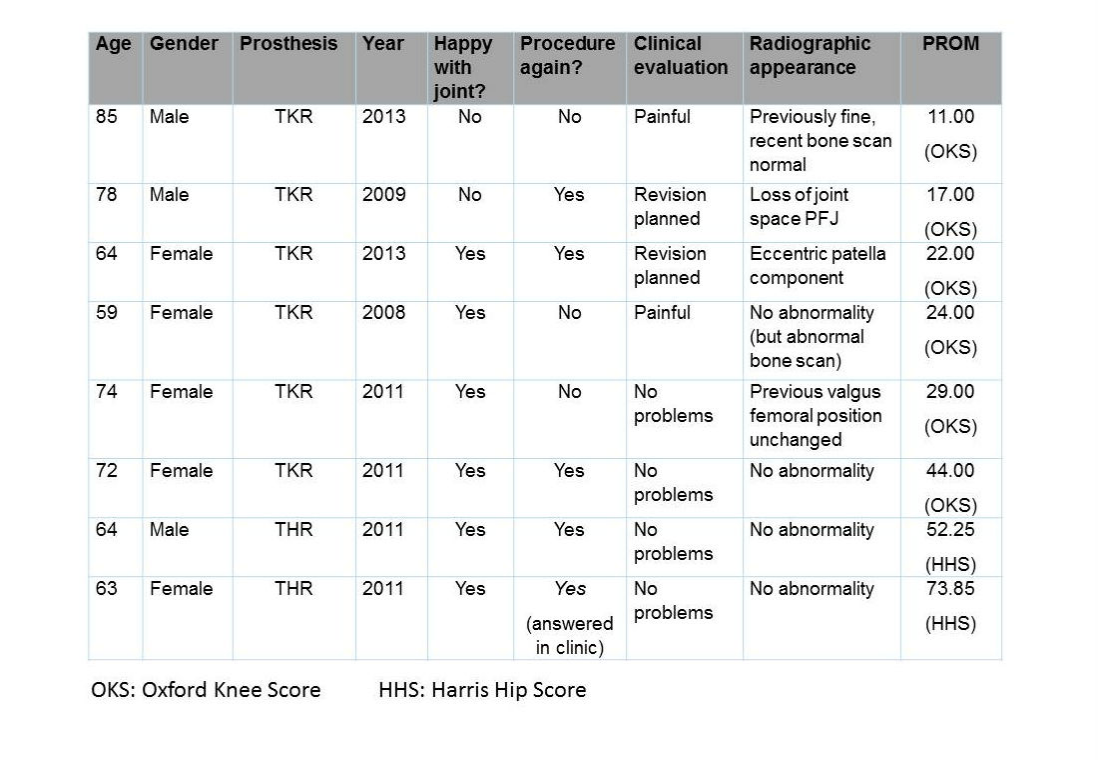
Summary of engaged and clinically assessed patient results.

A total of 8 patients engaged with text messaging, attended clinic, and completed clinical evaluation, radiological assessment, and PROM scores. The average age was 70 years (range 59-85 years). The results are shown in Figure 1. The patient who did not answer the second question stated that it would have been yes, but as she was attending clinic she preferred “to tell you in person.”

The typical values chosen by surgeons for the existing PROMs were an OKS of 24 or less (out of a total of 48) and an HHS of less than 70 (out of 100) as indicating a poor score needing surgical opinion [19]. Both of these values are subjective, decided by the surgeons’ experience. The table shows that 4 binary VSF results (positive or negative) matched the clinical evaluation and in 3 cases related to the existing PROM. In 1 case, a positive VSF result did not match (a patient planned for revision surgery); 3 of 4 mixed responses did not match clinical or radiographic findings but had concerning PROM scores.

**Figure 2:**
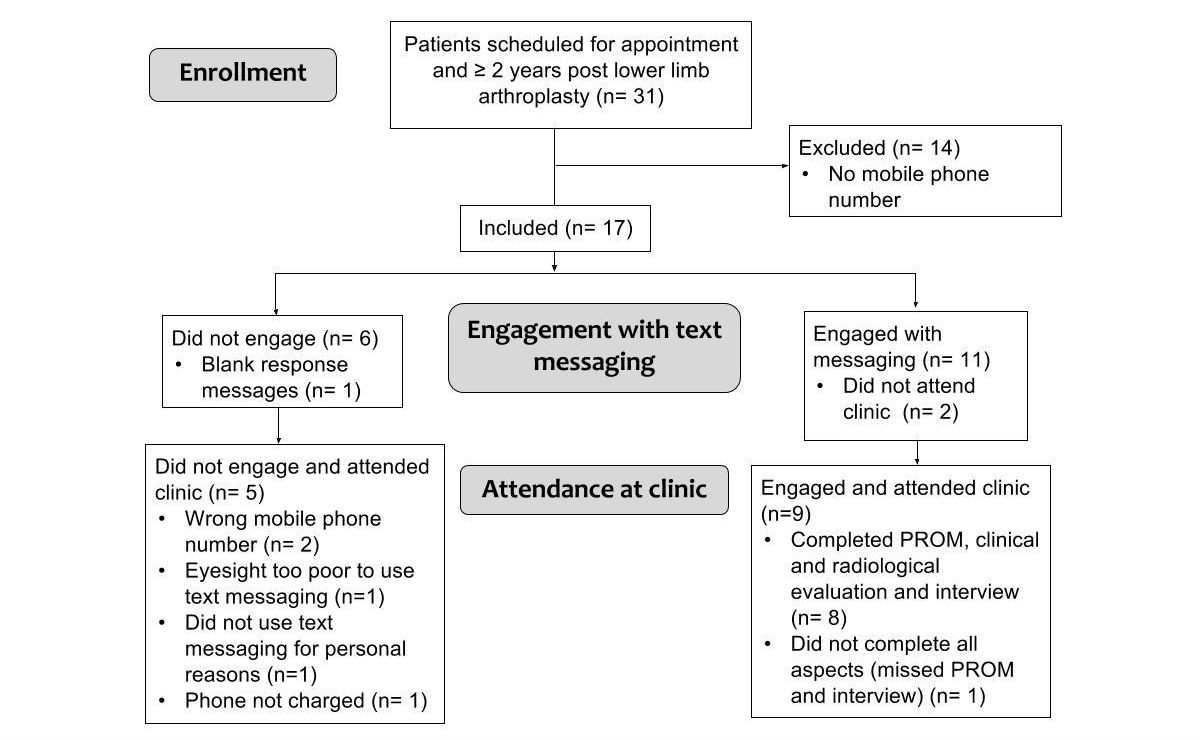
Study participation flow diagram.

### Patient Views

The 5 patients who did not engage with messaging were questioned on their reasons for not responding, and the reasons are summarized in Figure 2. The 8 patients who engaged with the text messages were asked the questions as outlined in Textbox 1. The transcribed recordings were analyzed by OB.

### Opinions on Text Messaging

A total of 5 confirmed that it was appropriate to receive such messages, and 2 volunteered that it was a good idea. A total of 5 expressed surprise that they had received text messages from the Health Board, and 3 patients thought it may have been a scam; patients highlighted how important it was for a message sender to identify themselves. One suggested that instead of an unknown phone number they would prefer the message to be assigned a contact name, such as “(name of) Hospital.” However, the clarity of the first message was praised for having identifiable names and knowledge of their surgeon and procedure.

### Views on the Oswestry Very Short Form

A total of 2 patients praised the VSF for being simple, and 2 said they would be happy to answer more questions by text message. Although all liked the brevity of the VSF, 5 patients wanted to expand on their answers, and 2 patients did provide additional information they thought was useful.

### Future Text Messaging

A total of 6 patients said they would be happy to receive further text messages from CAVUHB. The same number would be happy to communicate with their surgeon by text message if they were unable to see them in person, and 2 patients volunteered they would be happy to communicate with other members of the team instead of their surgeon. While 2 patients expressed a preference for communicating in future on a land line, 1 patient thought that text messaging would be “a good way for the NHS to save money.”

A total of 2 patients said they would not be happy using email to communicate in the future, and 2 would be happy to “touch base” using text messages on an annual basis, especially when there were unexpected gaps between appointments.

...on another day like this it could say “Have you any problems”....”no everything is fine, or great” and “I would be in touch with you in the future.”

Finally, 2 patients expressed dismay at the time spent attending or the distance travelled to an outpatient clinic when there were no problems.

There is your afternoon or your morning gone for the sake of 2 minutes. But if it was just a matter of a text message, it is so much easier.

## Discussion

### Principal Findings

Appreciating the limitations of this study is an important part of its message. This was pilot study with a small sample to meet the requirements of ethical approval under Continuing Service Improvement. The Health Board does not routinely follow arthroplasty patients up in outpatient clinic more than 1 year postoperatively; our population of patients was more than 2 years after arthroplasty. The Health Board agreement was for a small scale pilot study only as prior patient consent had not been obtained; the patients were not informed of this study from the start of their surgical experience, which may have affected their opinions and engagement. Encouragingly, of the patients who did not respond, none refused to participate. Reasons for not engaging related to wrong numbers and physical or personal reasons related to mobile use more generally.

The methodology was pragmatic, sampling within the confines of existing patient clinics. Further work requires patients to be identified, consented, and involved from the outset of the study. This project focused on the patient-doctor communication and primarily the patients’ views on text messaging. We have not considered other surgeons’ views apart from those of the authors (who by definition are biased favorably toward the project). Investigation into other orthopedic surgeons’ views, as well as those of administration staff who may be involved in the response and analysis of the text message communication, are required. Data security would need to be improved in the future (in line with Good Clinical Practice guidelines [20]). Texting patients sequentially from a smartphone is impractical for a large population as is transposing data from a survey tool to a spreadsheet. An ideal system would be a text messaging system allowing identification of patients, automatic sending and responding, and data capture with analysis and auditing. We suggest including applicable elements of the Checklist for Reporting Results of Internet E-Surveys, an existing matrix for designing Web-based surveys [21].

Our results show more than half of long-term arthroplasty patients have a mobile device and reported that they regularly used it. Of those, nearly two-thirds readily engaged with the text messaging intervention. In studies using Web-based surveys with orthopedic patients, less than half of patients (who had enrolled at the start of their patient experience) responded. In comparison, response rates in our study suggest text messaging may be a feasible option for long-term follow-up for at least a proportion of patients. This we believe could readily increase with better records of mobile numbers and increasingly prevalence in use of devices [22]. Texting is a limited format but short messages are attractive. There were conflicting opinions regarding communication via email and telephone. It is unlikely that there is a single method acceptable to all patients, and assumptions should not be made [13].

This was the first time the Oswestry VSF has been described in practice. The measure requires further validation as a clinical tool, which is beyond the scope of this paper. It was chosen specifically for its relevance, brevity, and ease of response. Patients made positive comments about its short length, although the results suggest it may be too brief for this group of patients. While some patients may be willing to complete an OKS by text message, the ideal orthopedic PROM for text message responses has yet to be devised. A text-based PROM should be designed to be useable by a patient who may have poor eyesight and arthritic fingers, who may take 25 minutes to read and respond to a single text message (as one patient described).

Of 4 positive VSF outcomes, 3 matched clinical and radiological findings. The one negative PROM matched clinical findings. PROMs delivered via mobile technology may play a useful role in filtering patients into groups: for example, satisfied patients who can be safely observed remotely, and unsatisfied patients who need clinical attention potentially more quickly than in existing outpatient formats. The problem, as seen from these small numbers, is that nearly half of the patients cannot be easily classified by the mobile PROMs. One author (JR) uses existing PROMs preoperatively and postoperatively to monitor trends in patient symptoms and satisfaction. The literature suggests that it is more useful to monitor the change in score than absolute values and that PROMs are more likely to identify satisfied patients [23]. Combining PROMs delivered by mobile technology with automated radiography reporting could allow trends to be monitored and changes correlated with radiographic findings. A combination of mobile messaging and radiographs has potential as a long-term, low-cost follow-up system for a large proportion of arthroplasty patients.

### Considerations for Future Research

As a result of our pilot study, we suggest future research into mobile technology delivered PROMs should do the following:

- Ensure engagement of the surgeon, hospital, and patient from the start of the process (preoperative assessment and patient education) to encourage participation, agree on a suitable communication method, gain consent, and ensure the probity of patient data
- Maintain accurate records of patient preferred contact details, which may not be limited to mobile or landline telephone numbers and email addresses. Patients, surgeons, and hospitals have a responsibility to keep patient information up to date and to use it to communicate effectively
- Consider physical barriers to using mobile technology and accept that such technology may not be suitable for all patients

### Conclusions

This pilot study on the use of text messaging to deliver PROMs to patients is an important first step to conducting rigorous research into new ways of monitoring outcomes in the long-term arthroplasty follow-up. Mobile technology, which is readily embraced by the arthroplasty demographic of today and could be universally used by the patients of tomorrow, should be engaged with and used by orthopedic surgery in the NHS. We have shown that many patients are willing and able to engage with mobile technology–delivered PROMs. Patient opinions on text messaging as a form of communication with their surgeons are generally positive, and text messaging could form an acceptable part of patient follow-up. For future research, we emphasize the importance of the involvement and engagement of patients and hospitals in these systems from the start of the arthroplasty pathway.

## Abbreviations

CAVUHB: Cardiff and Vale University Health Board
CAVOC: Cardiff and Vale Orthopaedic Centre
HHS: Harris Hip Score
NHS: National Health Service
OKS: Oxford Knee Score
PROMs: Patient Reported Outcome Measures
SMS: short message service
VSF: Oswestry Very Short Form
